# Application of the Yamaguchi criteria for classification of “suspected” systemic juvenile idiopathic arthritis (sJIA)

**DOI:** 10.1186/1546-0096-10-40

**Published:** 2012-11-23

**Authors:** Sharath Kumar, Divya Shree Kunhiraman, Lalitha Rajam

**Affiliations:** 1 Department of Pediatrics, Amrita Institute of Medical Sciences, Amrita Vishwa Vidyapeetham University, Cochin, India; 2 Department of Psychiatry, Amrita Institute of Medical Sciences, Amrita Vishwa Vidyapeetham University, Cochin, India; 3 Department of Rheumatology, ISIC hospital Sector-C, Vasant Kunj, New Delhi 110070, India

**Keywords:** Diagnosis, Arthritis, Juvenile idiopathic, Criteria, Adult-Onset Still’s disease, sJIA, Systemic JIA

## Abstract

**Background:**

Many children with sJIA may have a delayed onset of arthritis and so fail to fulfil the ILAR criteria for sJIA. This study was undertaken to determine whether the Yamaguchi criteria (for adult onset Still’s disease) is useful in classification of children with systemic juvenile idiopathic arthritis (sJIA) particularly in “pre-arthritic”, pure systemic, phase of the illness. A secondary objective was to determine the time delay between disease onset and onset of arthritis in our sJIA cohort.

**Methods:**

Retrospective chart review all patients with a diagnosis of systemic juvenile arthritis in our department from Jan 1, 2004 to Jan 1, 2010.

**Results:**

Twenty boys and eleven girls formed the study cohort. Thirteen patients were diagnosed with “suspected” sJIA due to typical systemic features but an absence of arthritis. Overall, the Yamaguchi criteria was fulfilled in a higher number of patients in the study (n=23) as compared to the ILAR criteria (n=18). Among the 13 “suspected” sJIA patients, 12 fulfilled the Yamaguchi criteria. Overall, either ILAR criteria or Yamaguchi criteria was fulfilled in 30 patients (96.8% of patients). The degree of association between the two criteria was poor (Phi coefficient = -0.352, p=0.05). Eleven out of eighteen patients with arthritis gave a history of delay in onset of arthritis (range=15 days to more than a year; median=30 days). Thus a total of 24 patients (75%) had a delay in onset of arthritis at onset of disease.

**Conclusion:**

Patients with sJIA can have a significant period during their course (particularly at onset) when they do not have arthritis. The Yamaguchi criteria may be useful in this subset of patients in the “pre-arthritic” phase of the disease. Future criteria should incorporate the strengths of both, the Yamaguchi and the ILAR criteria.

## Background

In comparison to the other diseases which are jointly grouped under the term juvenile idiopathic arthritis (JIA), systemic juvenile idiopathic arthritis (sJIA or Still’s disease as it was previously known) has many distinct features [1]. One important difference is that, at least in a subset of sJIA patients, arthritis may be a non-prominent and/or a delayed manifestation. Patients have been reported to have a delay of up to 10 years before the onset of arthritis [2].The International League Against Rheumatism (ILAR) criteria require the presence of arthritis to classify a patient as having sJIA [3]. Since morbidity and mortality may develop early in sJIA patients [2], a strict adherence to the ILAR criteria may lead to an unacceptable delay in diagnosis. Recent research in a small cohort from a single centre has demonstrated that sJIA patients prior to the onset of arthritis (“suspected sJIA”) appeared clinically and immunologically similar to patients fulfilling the ILAR criteria for sJIA [4].

Still’s disease (a.k.a. sJIA) is also known to occur in adults, in whom the disease is labeled as Adult Onset Still’s Disease (AOSD). Most studies comparing AOSD and Still’s disease in children have not found significant differences in the clinical features at presentation [5-9]. None of the criteria available for the diagnosis of AOSD depend heavily on the presence of arthritis. Among the different criteria available for the classification of AOSD, the Yamaguchi criteria are the most sensitive (96%) [10] and still manage to retain a high degree of specificity (92%) [11]. The Yamaguchi criteria have been compared to the ILAR criteria in Table 1[3,10]. The present study was undertaken in an attempt to determine the utility of the Yamaguchi criteria in the classification of children presenting with typical systemic features of sJIA who had still not developed arthritis (labeled as “suspected” sJIA). We also wished to determine the time lag between onset of fever and onset of arthritis among sJIA patients in our cohort who had fulfilled the ILAR criteria at presentation to our hospital.

**Table 1:** Comparison of the features of the ILAR and Yamaguchi criteria

**ILAR criteria**[3]	**Yamaguchi criteria**[10]
Arthritis in one or more joints with, or preceded by Daily fever of at least 2 weeks duration, that is documented to be quotidian for at least 3 days, and accompanied by one or more of the following	Major criteria
1. Evanescent (non-fixed) erythematous rash	1) Fever of 39C or higher lasting 1 week or longer
2. Generalized lymph node enlargement	2) Arthralgia lasting 2 weeks or longer
3. Hepatomegaly and/or splenomegaly	3) Typical rash $
4. Serositis	4) Leukocytosis (10,000/cmm or greater) including 80% or more of granulocytes
	Minor criteria
	1.) Sore throat
	2. ) Lymphadenoapthy and/or splenomegaly
	3.) Liver dysfunction
	4.) Negative Rheumatoid Factor (RF) and negative Antinuclear antibody (ANA) test
	Diagnosis is made when there are 5 or more criteria which include atleast 2 major criteria.
Exclusions:-	Exclusions
a. Psoriasis or a history of psoriasis in the patient or first degree relative	1.) Infections
b. Arthritis in an HLA B27-positive male beginning after the 6th birthday	2.) Malignancies
c. Ankylosing spondylitis, enthesitis related arthritis, sacroiliitis with inflammatory bowel disease, Reiter’s syndrome, or acute anterior uveitis, or a history of one of these disorders in a first degree relative	3.) Rheumatic diseases
d. The presence of IgM rheumatoid factor on at least 2 occasions at least 3 months apart.	

$ Typical rash = Nonpruritic macular or maculopapular salmon colored rash (usually over trunk or extremities while febrile).

## Methods

The study was a retrospective chart review conducted in the pediatrics department of a tertiary level teaching hospital. Electronic medical records were mined using key words “arthritis”, “JIA” and “JRA”. All patients with these keywords mentioned anywhere in their notes, and had been diagnosed between Jan 1, 2004 and Jan 1, 2010 were identified. Their detailed case records were reviewed and those patients finally diagnosed by the treating pediatrician (SK, LR and others) as “definite” sJIA (fulfilling the ILAR classification criteria) or “suspected” sJIA (not fulfilling the ILAR criteria) were included. Accuracy of diagnosis was confirmed by the first author (SK). For inclusion in the cohort, the patients had to have been investigated at length to rule out infections and malignancies as causes of fever of unknown origin (FUO).

The chart review was conducted independently by two of the authors (SK and DKR). Any differences were resolved by consensus. The fulfillment of the various variables in both the Yamaguchi criteria and the ILAR criteria were identified in each patient at the time of first presentation to our institute. Exclusionary components of the criteria set were also considered. (Refer Table 1 for the criteria) Variables representing symptoms (characteristic fever, arthralgia, and sore throat,) were considered positive irrespective of whether they had it at presentation to our institute or at any time in the past during the course of the disease.

Laboratory criteria (leukocyte count, liver function test, serositis and negative ANA, RF) and clinical signs (typical rash, splenomegaly, hepatomegaly, and lymphadenopathy) were considered positive only if they had been performed/observed in our department at the time of presentation and had been fulfilled. For the presence of arthritis, either physician documentation at presentation, or patient/parent report were considered sufficient to be graded positive. However, in case of parent/patient reported arthritis, the child was considered to have had arthritis only if in addition to arthralgia the patient had had either significant swelling which had been documented by a doctor or any 2 of the following physical findings: limitation of range of motion/deformity, pain on motion leading to limitation of motion or increased warmth over the joint.

The time duration between onset of fever and onset of arthritis as reported by the patients or their parents (based on information available in the charts or information obtained via a telephonic interview) was also recorded. The statistical significance of any difference was inferred using the Fisher test for nominal values and the independent t test for continuous variables. The coefficient of association between the criteria was calculated using the Phi coefficient. Approval for the study was obtained from the Institutional Ethics Committee.

## Results

Thirty-four patients were diagnosed with sJIA (definite or suspected) in the last 6 years. Three patients (all girls) were excluded due to insufficient data in their medical records. Twenty boys and eleven girls (n=31) formed the study cohort. All of the patients were DMARD naïve at presentation and none had received steroids for more than 2 weeks. Thirteen patients failed to fulfill the ILAR criteria, all of them due to a lack of arthritis. The mean age of children who fulfilled the ILAR criteria (11.9 years) was significantly higher than patients who did not fulfill the ILAR criteria (6.9 years, p=0.006) (Table 2).

**Table 2:** Characteristics of the cohort comprising sJIA and “suspected” sJIA cohort

**Characteristics**	**sJIA (ie. fulfilling ILAR criteria)**	**“suspected” sJIA**	**Total cohort**
	**n = 18**	**n =13**	**n = 31**
Mean age (years)	11.9	6.9**	9.8
Male:Female	13:5	7:6NS	20:11
Mean duration of symptoms at time of presentation (days)	109.1	51.8NS	85.1
Number of patients who received pre treatment with steroids	8(44.4%)	2(15.4%)NS	10
Mean daily dose of steroids (mg/kg/day)#	0.96	2.47NS	1.3
Mean number of days that the patients had received steroids	8.13	7.5NS	8
Number of patients who developed MAS on follow up	1	6*	7
Average number of joints	9	0*	5.2
Mean ESR(mm/hr)	62.2	43.8**	54.5
Mean CRP (mg/L)	130.5	116.2 NS	124.2
Mean Hemoglobin (gm/dL)	10.9	10.2 NS	10.6
Mean WBC (cells x 10^9^/L)	15.2	20.7 NS	17.5
Mean platelet count (cells x 10^9^/L)	337.6	468.4 NS	413.6
Mean ALT (U/L)	62.4	107.9 NS	81.5
Mean AST (U/L)	55.5	41.3 NS	49.5

*= p value <0.05, ** = p value < 0.005 ,NS = not significant.

# Mean value only of those patients who had been treated with steroids.

Among the 13 patients who did not fulfill the ILAR criteria, nearly all children (12 patients) fulfilled the Yamaguchi criteria. Among the 18 patients who fulfilled the ILAR criteria, 7 patients did not fulfill the Yamaguchi criteria (Table 3). The association between a fulfillment of ILAR criteria and fulfillment of Yamaguchi criteria was evaluated. The Phi coefficient was -0.352 (p=0.05). Thus the total number of patients who fulfilled the Yamaguchi criteria was 23 (74%), compared to only 18 patients (58%) who fulfilled the ILAR criteria.

**Table 3:** The number of patients who fulfilled the Yamaguchi criteria and the ILAR criteria

	**ILAR criteria fulfilled**	**ILAR criteria not fulfilled**	**Total**
Yamaguchi criteria fulfilled	11	12	23
Yamaguchi criteria not fulfilled	7	1	8
Total	18	13	

**Figure 1:**
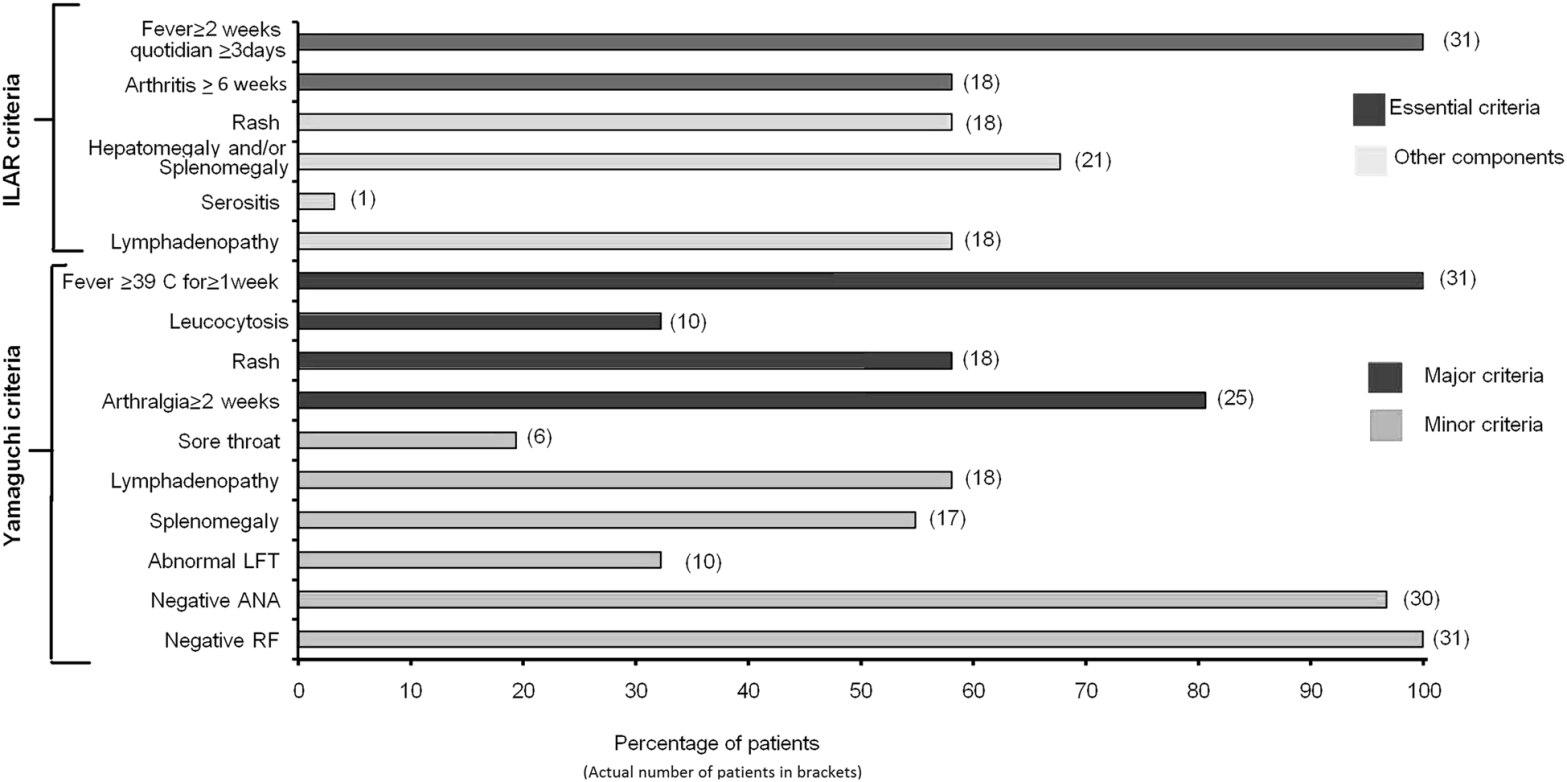
**The percentage of patients fulfilling the different component criterion of the ILAR and Yamaguchi criteria in our cohort.** Actual numbers of patients are in brackets.

The exclusionary components of both the ILAR and the Yamaguchi criteria were absent in all of the patients. The number of patients fulfilling the different components of the ILAR criteria and the Yamaguchi criteria are shown in the Figure 1. All of the patients fulfilled both the Yamaguchi criteria component as well as the ILAR criteria component for fever. The second most commonly fulfilled component was “Arthralgia> 2 weeks” (Yamaguchi criteria) which was seen in 28 patients (which included patients with arthritis as well). In contrast only 18 out of 31 patients had arthritis at presentation.

A comparison of the baseline lab characteristics of patients who fulfilled the ILAR criteria and those with “suspected” sJIA has been shown in Table 2. The difference in characteristics was tested using the Fisher’s exact t test for nominal values and the independent t test for continuous variables. During the initial disease presentation, all patients, in addition to a detailed evaluation for an infectious cause, had undergone a bone marrow aspiration. The bone marrow study had been reported as normal in every child.

A higher proportion of patients who fulfilled the ILAR criteria had received steroids, though the mean dosage of steroids (among patients who had received them) was higher in the “suspected” sJIA group. Among the children who had received steroids, (n=10), 8 patients had arthritis and 6 had the typical sJIA rash. In comparison, among patients who had not received steroids (n=21), 10 patients had arthritis and 12 patients had typical rash.

Among the 13 patients with “suspected” sJIA, 6 patients had neither arthralgia nor arthritis and 7 patients had arthralgia without ever having had arthritis. The presence of various clinical features in patients grouped according to the presence or absence of arthritis and arthralgia is shown in Table 4. On comparison none of the differences between the groups were statistically significant.

**Table 4:** The number of patients with various clinical features divided according to the presence of arthritis and arthralgia

**Components of Yamaguchi criteria (Y) or ILAR criteria (I)**	**Arthritis absent (i.e. “suspected” SoJIA) n=13**		**Arthritis present (i.e. “definite” SoJIA) n=18**
	**Arthralgia absent n=6**	**Arthalgia present n=7**	
	**n (% of pts without arthralgia)**	**n (% of patients with arthralgia)**	**n (% of patients with arthritis)**
Rash (Y,I)	5 (83)	3 (43)	10 (56)
WBC (Y)	1 (17)	3 (43)	6 (33)
Sore throat (Y)	1 (17)	2 (29)	3 (17)
Lymphadenopathy (Y,I)	5(83)	4 (57)	9 (50)
Abnormal LFT (Y)	3 (50)	4 (57)	3 (17)
Splenomegaly (Y)	5 (83)	5 (71)	7 (39)
Hepatomegaly OR splenomegaly (I)	5 (83)	6 (86)	10 (56)
Only splenomegaly (N)	2 (33)	3 (43)	1 (6)
Splenomegaly AND Hepatomegaly (N)	3 (50)	2 (29)	6 (33)
Yamaguchi criteria	5 (83)	7 (100)	11 (61)

Y = Component of Yamaguchi criteria; I = Component of the ILAR criteria ; N = Not a component of either criteria. WBC = Leukocytosis (10,000/cmm or greater) including 80% or more of granulocytes, LFT = Liver function tests.

All patients with “suspected” sJIA, after extensive evaluation for infection and malignancy, received treatment with systemic corticosteroids for more than a month for the persistent and unexplained systemic inflammatory illness. During followup (until the end of the study), 3 patients with “suspected” sJIA developed arthritis. One patient developed a transient arthritis a month after initial presentation. The arthritis was present in a single joint and lasted for 3 days. The other 2 patients developed a persistent oligoarthritis. One child developed 2 active joints and the second patient develop arthritis in 4 joints. The delay between first presentation and development of arthritis was 3 months and 7 ½ months, respectively.

**Figure 2:**
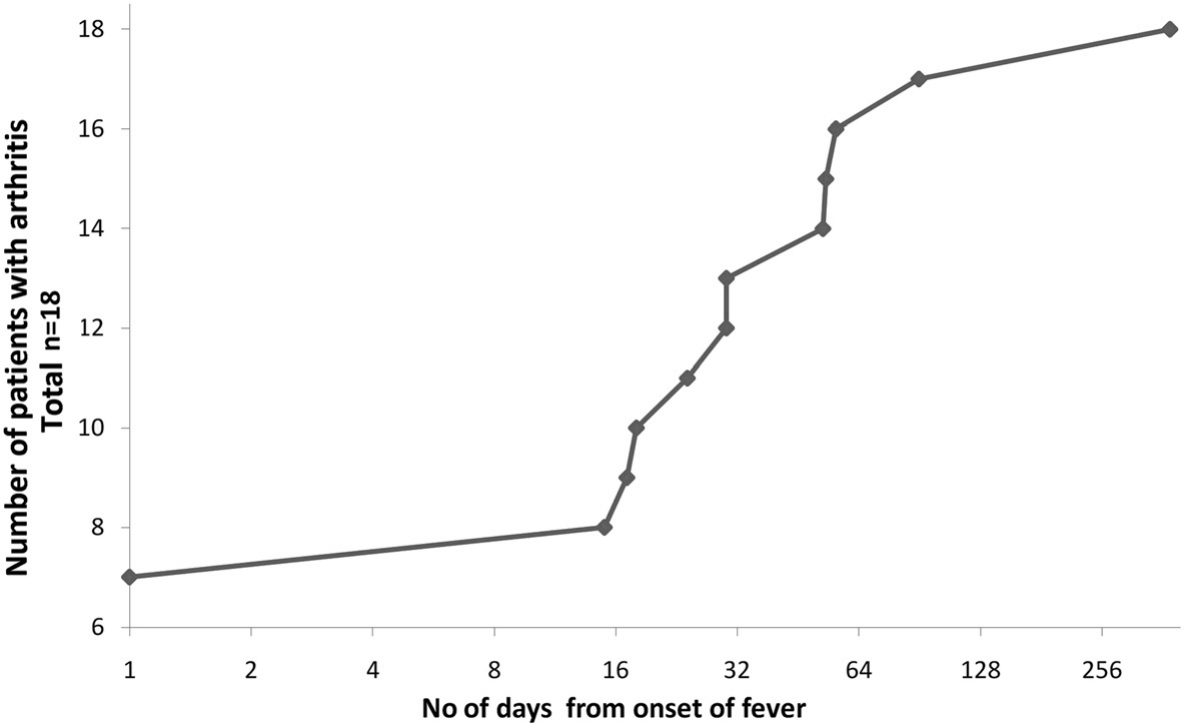
**Figure depicting the delay between onset of fever and onset of arthritis in the eighteen patients who fulfilled ILAR criteria at presentation to our department.** Cumulative number of patients with arthritis is plotted on the y axis against the number of days (in geometric progression) since they developed fever on the x-axis.

Each of the18 patients who had arthritis at presentation fulfilled the ILAR criteria. However, upon careful interviewing it was revealed that 11 of these patients had a history of delay between onset of systemic features and onset of their arthritis. The duration of the delay varied from 15 days to more than a year [median=30 days; mean +/- (SD) =69.3 days +/- 104.6 days]. The delay between the onset of fever and the onset of arthritis in these 11 patients is depicted in Figure 2.

**Figure 3:**
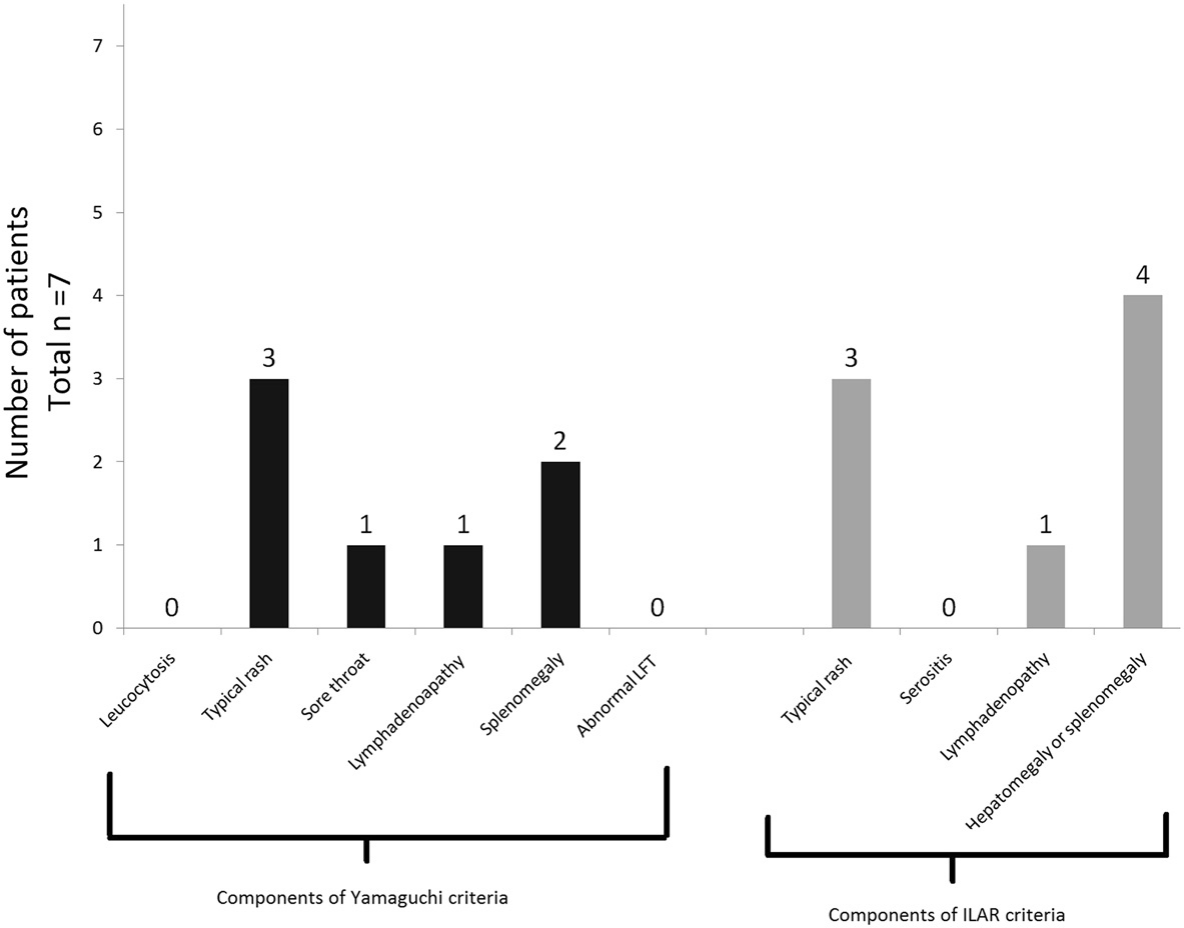
**Characteristics of patients who fulfilled the ILAR criteria but failed to fulfil the Yamaguchi criteria.** (Total n=7) All patients had fever and arthritis.(components of both Yamaguchi and ILAR criteria). None of the patients had a positive ANA nor RF (Component of Yamaguchi criteria). Further components of Yamaguchi criteria are in black and of the ILAR criteria are in grey.

Characteristics of the patients who fulfilled the ILAR criteria but failed to fulfill the Yamaguchi criteria are depicted in Figure 3. All seven patients had fever and arthritis. All patients had a negative ANA as well as a negative RF. Thus they required the presence of another 2 components for fulfillment of the Yamaguchi criteria. However none of the patients fulfilled more than one additional component.

## Discussion

The change in terminology from Still’s disease to sJIA described in the ILAR criteria leads to situations where children clearly have this disease, but are unable to be classified as sJIA due to the absence of arthritis. Since the ILAR classification criteria were published in 2004, there has been extensive research on the pathogenic mechanisms involved in sJIA. The 2 most important findings, i.e. , the role of interleukin-1 beta and interleukin-6 in sJIA, has translated not only into a better understanding of the disease but has also lead to the use and approval of newer and possibly more effective therapeutic agents (anakinra and tocilizumab respectively) [12,13].

Basic research has continued to uncover other cytokines and biomarkers which are characteristically increased in sJIA. These include myeloid-related proteins 8 and 14 (MRP 8/14) [14-16], interleukin-18 (IL-18) [17-19], macrophage migration inhibitory factor (MIF) [20,21], S100A12 protein [22], soluble IL-2 receptor [23], NK cell function [24], and others. These biomarkers have been studied regarding their utility in diagnosing patients with sJIA, both individually [16,18,19,21,22] and as part of a panel [18,25]. Initial results have been quite promising.These biomarkers may also be helpful in distinguishing children with systemic JIA from patients suffering from either other subtypes of JIA, other inflammatory diseases (like Kawasaki disease) or from infections. The baseline clinical characteristics, the mean value of many of the above biomarkers , and the response to therapy of 5 “suspected” sJIA patients without arthritis were described in the work of Vastert et al. [4]. These “suspected” sJIA patients had baseline clinical characteristics and mean value of many of the above mentioned biomarkers similar to a comparison group of 15 sJIA patients fulfilling the ILAR criteria. In addition, the “suspected” sJIA also had a comparably robust improvement on anakinra. Unfortunately, these biomarkers are not routinely available in clinical practice. On the other hand, the Yamaguchi criteria for AOSD are a relatively simple tool with clinical and basic laboratory features [10].

Including the seminal publication of Cabane et al [5] in 1990, there have been 6 publications [6-9,26] comparing children and adults with Still’s disease. Most of these studies have revealed a lack of any significant difference between the two groups with respect to the systemic features [5-9], the articular manifestations, or sequelae [5,7-9]. However, a few significant differences between pediatric and adult patients have been pointed out by Pay et al. with respect to clinical and laboratory features [26]. Lin et al. has also noted differences between AOSD adults and sJIA children in articular outcomes [6].

Pay et al. showed significant differences with respect to the frequency of fever, rash, myalgia, weight loss, sore throat, LFT abnormalities and neutrophilia which were all higher in patients with AOSD. The pattern of joint involvement was also slightly different. Patients with sJIA had a higher rate of involvement of knee, ankle, elbow, metatarsophalangeal joints, hip joints as well as the cervical spine. However, in spite of these differences, given the large number of similarities the authors conclude that AOSD and sJIA may still be the same disease, and children may simply be reacting differently [26]. This may be true given the fact that, with the exception of this one publication (Pay et al. [26]), the other five publications revealed a lack of any significant difference between the two groups with respect to the systemic features or the articular manifestations at presentation [5-9].

Thus, it appears to be a widely-held belief that sJIA and AOSD are the same disease, albeit with slight differences. Given the similarities, it would be reasonable to expect that sJIA patients would fulfill diagnostic criteria for AOSD. In fact, in the study by Luthi et al as well, all 9 of their sJIA patients fulfilled the Yamaguchi criteria [7]. Thus the fact that the Yamaguchi criteria were fulfilled in 74% of patients in our cohort was not as surprising as the fact that only 58% of patients fulfilled the ILAR criteria for sJIA. The performance of the ILAR criteria in the present cohort was comparable to that reported in a previous cohort from a multicenter registry in the state of Pennsylvania, USA (PASOJAR cohort). In the PASOJAR cohort, only 31% of patients (42/136 sJIA patients) fulfilled the ILAR criteria [27]. The failure of ILAR criteria in our cohort was mainly due to the absence of arthritis.

Data pertaining to the delay in (or absence of) arthritic manifestations of sJIA is scarce. A cohort of 46 patients with “probable” Still’s disease (as defined by the Taplow group criteria) was published in 1962. These children were followed for a mean period of 5.9 years. Ten of these patients who did not evolve into any disease had unique features and were labeled as “benign systemic disease” due to the fact that all had fever with rash but none had persistent arthritis. Arthralgia was present in all of them and 7 of them had arthritis lasting only 2 to 3 days [28]. In the PASOJAR cohort, it was reported that 12% of their patients did not have arthritis [27]. Even in the study by Pay et al, in which all sJIA patients ultimately fulfilled the ILAR criteria, 5/25 (20%) sJIA patients did not have arthritis at initial presentation. The frequency of arthritis or arthralgia was not significantly different between sJIA and AOSD patient groups in the same cohort [26]. Uppal et al reported that 30% of their patients with juvenile onset Still’s disease did not have arthritis and of these only 17% of patients had arthralgia [9]. These numbers are lower than what was found in our cohort. This could possibly be presumed to be due to the fact that patients without arthritis are more likely to be referred to a tertiary level hospital.

As part of the effort to develop consensus based treatment plans and a standardized assessment schedule for sJIA, the Childhood Arthritis and Rheumatology Research Alliance (CARRA) in the US conducted a case-based online survey prior to a consensus meeting. Among the 63 pediatric rheumatologists who completed the survey the majority (87.9%) found the initiation of treatment in sJIA acceptable in the absence of arthritis based on fever and systemic features such as characteristic rash, serositis, and adenopathy, provided that infection and malignancy had been excluded. The final operational definition of sJIA developed by CARRA for initiating treatment in patients was different from the ILAR classification criteria for sJIA. The opinion of the consensus group was that the ILAR criteria were “too stringent” and might often lead to exclusion of patients who need to be treated as sJIA [29]. Despite fulfilling the apparently more stringent ILAR criteria, 7 patients in our cohort failed to fulfill the Yamaguchi criteria. The components of the two criteria fulfilled by these patients are shown in Figure 3. In comparison to the entire cohort, the ILAR criteria appeared to be more sensitive in patients with arthritis. The ILAR criteria require the presence of any one extra feature (apart from fever and arthritis) for fulfillment of the classification criteria. However, in the same patients, three additional features (apart from fever and arthralgia due to arthritis) were required to fulfill the Yamaguchi classification criteria. It appears that in patients with arthritis the "ILAR criteria are more commonly fulfilled (100%) than the Yamaguchi criteria (61%). The Yamaguchi criteria seem to be more efficacious in the absence of arthritis (12/13) compared to patients with arthritis (11/18) (Table 4). It is also interesting to note that one patient was diagnosed as “suspected” sJIA based on splenomegaly, typical fever along with the typical sJIA rash.

One of the features typical of sJIA is the characteristic evanescent rash [2,29]. The prevalence of rash in previous sJIA cohorts reported from Europe or North America has been quite high. (~80%) [27,30]. Previous JIA cohorts from India have demonstrated that the prevalence of rash, in Indian sJIA patients, vary between 27 to 57% [31,32]. Similarly, in our cohort only 18 patients (58%) had the typical rash associated with sJIA. The lower incidence of rash in studies in India may be due to darker skin color that makes the rash harder to see. This difficulty may represent a further limitation of the ILAR criteria in ethnicities associated with darker skin colors.

The ILAR criteria for JIA have been criticized on various counts. One of the criticisms has been the lack of consideration or integration of adult criteria for diseases, like Still’s disease, psoriatic arthritis and spondyloarthropathy, which occur in both children and adults. The utility of such integration was tested in a single cohort of 683 JIA patients in whom 157 patients had been grouped under undifferentiated as per the ILAR (Durban) criteria. A hierarchal tree approach with use of validated adult criteria reduced the number of patients in the undifferentiated category to zero [33]. Analogous to this, in our cohort, when both criteria (ILAR criteria and Yamaguchi criteria) were used in an “either/or” combination, nearly all the patients (30 out of 31) could be classified as sJIA.

During development of the CARRA consensus treatment plans for new-onset sJIA, there was extensive discussion over the fact that patients in the early part of sJIA do not fulfill the ILAR criteria (probably due to lack of arthritis) and “yet need treatment” [29]. The response of early pre-arthritic or “suspected” sJIA patients to anakinra in the study by Vastert et al., suggests that treatment of these “suspected” sJIA patients, may help us to utilize a window of opportunity, and possibly prevent some of the morbidity associated with sJIA [4]. 

There are many limitations to this study. The data was retrospectively obtained from a small cohort of patients diagnosed at a single centre. Being a tertiary level referral hospital, the cohort may not be representative of patients in the community. Telephone interviewing to recall the gap between arthritis and systemic features is a crude method at best. The lack of controls (other inflammatory diseases like Kawasaki disease or inflammatory bowel disease; malignancies and infections) prevents a determination of the specificity of the two criteria. Parts of the Yamaguchi criteria such as sore throat could probably have reduced specificity in children. The peak incidence of sJIA is between 1 and 5 years of age [34]. Children at this age are less likely to complain of “arthralgia” or “sore throat”. The patients in the cohort were investigated at length and infections and malignancies were ruled out as possible causes of their symptoms . However, none of the patients were investigated for autoinflammatory diseases which are known to mimic sJIA. Since the prevalence of autoinflammatory syndromes is quite low with only case reports from India, it is unlikely that patients with these disorders could have formed a considerable number in the cohort. Nevertheless, it is possible that patients fulfilling the Yamaguchi criteria alone may have had a distinct disease which is similar to sJIA but not the same. Another limitation was the fact that long-term followup data was not part of the present study. It is possible that patients in our cohort with suspected sJIA may evolve into other rheumatologic disorders on long-term follow up. In the cohort of “probable” sJIA by Ansell et al., 20 out of 43 patients evolved into another disease on followup. Thus it is prudent to point out that given all these above limitations, the results pertaining to the primary objective of the study should only be viewed as hypothesis-generating.

As is evident from the present cohort as well as previous cohorts, patients with sJIA are a heterogeneous group [35]. A subset of patients may not have arthritis for a significant period at the onset of the disease, but may still have elevated biomarkers associated with sJIA [4]. Some of these new biomarkers have been found to specific to sJIA and could represent future diagnostic markers [16,18,19,21,22,25]. Initial evidence seems to suggest that the subset of patients without arthritis, or with minimal arthritis, may respond to therapy equally well, or better, than patients with prominent joint involvement [4,35]. Modification of the ILAR criteria with inclusions of these biomarkers, would help in classification of sJIA patients without arthritis, enable them to qualify for enrollment in trials as well as become eligible for treatment. However, these tests are not yet available outside the research setting and are likely to be expensive when they do become available. In the meantime, the use of the Yamaguchi criteria, with suitable modifications, in a hierarchal fashion, along with the ILAR criteria (analogous to that used in a previous Italian cohort [33]), might prove to be a useful strategy to help classify “suspected” sJIA patients in resource poor settings.

## Conclusions

There is a subgroup of sJIA patients in whom arthritis is absent or quite delayed and in this group, the diagnosis may be very challenging. Comparison of the ILAR and Yamaguchi criteria in our cohort of sJIA patients suggests that some changes to the ILAR criteria for sJIA are probably necessary to make it more inclusive. Possible inclusion of recently described biomarkers or application of the Yamaguchi criteria (which do not consider arthritis as an essential feature), might enable the criteria to be more sensitive and clinically useful. These changes may facilitate early and timely diagnosis of children with sJIA and thus, allow early treatment.

## Abbreviations

AOSD: Adult onset still’s disease; DMARD: Disease modifying anti-rheumatic drugs; ILAR: International league of associations for rheumatology; FUO: Fever of unknown origin; sJIA: Systemic juvenile idiopathic arthritis.

## Competing interest

The authors had no conflicts of interest.

## Author contributions

SK developed the concept for the study, was involved in data collection and wrote and reviewed the manuscript. He had full access to all the data in the study and takes responsibility for the integrity of the data and the accuracy of the data analysis. DSK was involved in data collection and analysis and reviewed the manuscript. LR was also involved in conceptualizing the study, as well as in critical review of the manuscript. All authors read and approved the final manuscript.
